# 

**Published:** 1895-03

**Authors:** 


### CONTENTS.

COMMUNICATIONS.

Odontalgia .... 105
Characteristics .... 117
The Use of Sulphuric Acid in the Treatment of Roots for Immediate Filling .... 121
Some Peculiarities of Oxyphosphates .... 126

MONTHLY DIGEST.

Operative Dentistry .... 132

PROCEEDINGS.

First District Dental Society of New York—Clinic .... 149
Tri-State Meeting .... 153

EDITORIAL.

Mississippi Valley Dental Society .... 154
The Sanitarian .... 154
Biographical .... 155

